# Facilitation of Ovarian Response by Mechanical Force—Latest Insight on Fertility Improvement in Women with Poor Ovarian Response or Primary Ovarian Insufficiency

**DOI:** 10.3390/ijms241914751

**Published:** 2023-09-29

**Authors:** Chia Lin Chang

**Affiliations:** Department of Obstetrics and Gynecology, Chang Gung Memorial Hospital Linkou Medical Center, Chang Gung University, Guishan, Taoyuan 33305, Taiwan; amego@cgmh.org.tw; Tel: +886-975365869

**Keywords:** IVA, WOLI, poor ovarian response, primary ovarian insufficiency, laparoscopic incision, IVF

## Abstract

The decline in fertility in aging women, especially those with poor ovarian response (POR) or primary ovarian insufficiency (POI), is a major concern for modern IVF centers. Fertility treatments have traditionally relied on gonadotropin- and steroid-hormone-based IVF practices, but these methods have limitations, especially for women with aging ovaries. Researchers have been motivated to explore alternative approaches. Ovarian aging is a complicated process, and the deterioration of oocytes, follicular cells, the extracellular matrix (ECM), and the stromal compartment can all contribute to declining fertility. Adjunct interventions that involve the use of hormones, steroids, and cofactors and gamete engineering are two major research areas aimed to improve fertility in aging women. Additionally, mechanical procedures including the In Vitro Activation (IVA) procedure, which combines pharmacological activators and fragmentation of ovarian strips, and the Whole Ovary Laparoscopic Incision (WOLI) procedure that solely relies on mechanical manipulation in vivo have shown promising results in improving follicle growth and fertility in women with POR and POI. Advances in the use of mechanical procedures have brought exciting opportunities to improve fertility outcomes in aging women with POR or POI. While the lack of a comprehensive understanding of the molecular mechanisms that lead to fertility decline in aging women remains a major challenge for further improvement of mechanical-manipulation-based approaches, recent progress has provided a better view of how these procedures promote folliculogenesis in the fibrotic and avascular aging ovaries. In this review, we first provide a brief overview of the potential mechanisms that contribute to ovarian aging in POI and POR patients, followed by a discussion of measures that aim to improve ovarian folliculogenesis in aging women. At last, we discuss the likely mechanisms that contribute to the outcomes of IVA and WOLI procedures and potential future directions.

## 1. Introduction

### 1.1. The Root of Reduced Fertility in Aged Women

Infertility is a growing medical concern with ~15% of couples confronting fertility-related issues [1]. Due to delayed childbearing, over the last decade, there has been a 30% expansion in the utilization of medically aided reproduction, especially among women aged 40 or higher [1,2]. For women over 40 years of age, there was a 55% upsurge in the number of assisted reproductive technology (ART)-conceived birth in select country in the past decade [1]. Despite considerable progress in reproductive science, allowing more than 80% of couples experiencing infertility to conceive a child, clinicians in IVF centers are having difficulties overcoming the aging-ovary-related decline in fertility. The treatment of aging patients with poor ovarian response (POR) and reproductive-age women with primary ovarian insufficiency (POI) has become one of the foremost obstacles faced by contemporary in vitro fertilization (IVF) centers [3].

The incidence of POR rises dramatically with increasing age and is associated with a range of impaired ovarian functions [4]. The physiological changes in the ovary of POR patients that accompany aging include a reduction in follicle number, a decline in oocyte quality, and altered reproductive endocrinology [5]. On the other hand, POI is a complex disorder that is characterized by the depletion of primordial ovarian follicles and the associated physiological consequences [6,7,8,9]. POI affects ~1% of women and represents a severe end of the premature ovarian aging spectrum, in which menopause occurs before 40 years old.

In this review, we provide an overview of recent progress in the use of mechanical procedures to improve reproductive outcomes in women with POR or POI and potential mechanisms underlying these mechanical procedures. Because the age-related decline in reproductive capacity of POR and POI patients is primarily due to the depletion of follicle reserve and the deterioration of oocyte quality and ovarian environment [10], we will first provide a brief sketch of ovarian aging and potential treatments for averting this process, which include the mechanical-force-based procedures. This will be followed by a discussion of potential mechanisms that underlie mechanical-manipulation-induced stimulation of follicle development in patients with extremely poor ovarian response (EPOR) or resistant ovary syndrome.

### 1.2. A Myriad of Approaches Have Been Attempted to Prevent or Slow down the Process of Ovarian Aging

To address the issue of ovarian aging, preimplantation genetic diagnosis/screening (PGD/PGS) is used to examine the likelihood of aneuploidy, whereas ovarian reserve testing, oocyte selection and donation, and ovarian tissue cryopreservation are used to help improve fertility outcomes [11,12]. For the treatment of POR patients, personalized ovarian stimulation and enhanced embryo selection via genetic testing have been explored. In addition, adjunct treatments, including growth hormone, clomiphene, letrozole, estradiol, hCG, CoQ10, testosterone, dehydroepiandrosterone (DHEA), melatonin, resveratrol, metformin, dasatinib, quercetin, and sphingosine-1-phosphate analog, have been attempted to prevent or slow down the process of ovarian aging [11,13,14,15,16,17,18,19,20,21]. Research into gamete generation has also generated hope to produce viable embryos through in vitro production of mature oocytes using embryonic stem cells, induced pluripotent stem cells or primordial follicles for fertility treatment in the future [22,23,24]. Despite these efforts, the results have been inconsistent, and none have consistently improved fertility in aging IVF patients [25].

Given the limitations of gonadotropin- and steroid-hormone-based approaches in activating primordial follicles, novel pharmacological intervention that breaks follicle dormancy or facilitates early follicle growth has been viewed as a promising approach to improve IVF outcomes in aging women [26,27]. To this end, a plethora of studies have been conducted on pharmacological activators that target the phosphatidylinositol-3-kinase (PI3K)-PTEN (phosphatase and tensin homolog deleted from chromosome 10)-Akt-(protein kinase B)-Foxo3 pathway for early follicle development [27,28,29,30,31,32,33]. One of the most intriguing areas of investigation in this context is the In Vitro Activation (IVA) procedure, which combines pharmacological activators and mechanical perturbation of ovarian cortex tissues to initiate primary follicle development [28,34,35]. The procedure has generated significant interest and shows promising results in women with POR or POI. Additionally, recent studies have revealed that a laparoscopic incision-based procedure (i.e., whole ovary laparoscopic incision (WOLI) or laparoscopic ovarian incision (LOI)), which solely relies on mechanical manipulation to enhance follicle growth, has been successful in providing live births in certain IVF patient populations [28,36,37,38].

Despite recent progress, the lack of a comprehensive understanding of the complex molecular and mechanical interactions involved in the regulation of follicle growth and activation remains a major challenge to optimize these procedures and improve their clinical applicability. Nevertheless, the potential benefits of these approaches are clear, and they offer exciting opportunities to improve fertility outcomes in aging women with POR or POI.

## 2. Reproductive Characteristics of Aging IVF Patients: Ovarian Aging and Poor Ovarian Response

### 2.1. The Process of Ovarian Decay in Aging Women Is a Multi-Factorial Phenomenon

The aging in females is closely associated with a higher incidence of infertility and miscarriage and eventual onset of menopause, which occurs when the number of oocytes falls below a critical threshold [39,40,41,42]. As women age, the risk of poor ovarian response to traditional hormonal stimulation increases, as indicated by smaller ovarian volume, lower antral follicle count, and decreased ovarian stromal vascularity [43,44,45]. Studies of aged animals and women have shown that there is an inverse correlation between age and antral follicle count, the number of oocytes obtained after gonadotropin stimulation, and anti-Müllerian hormone (AMH) level, while age is positively correlated with basal FSH level [46,47,48,49]. In this context, AMH is considered a biomarker to predict the response to ovarian hyperstimulation and to assess ovarian aging and ovarian reserve [50,51,52,53,54,55].

**Figure 1:**
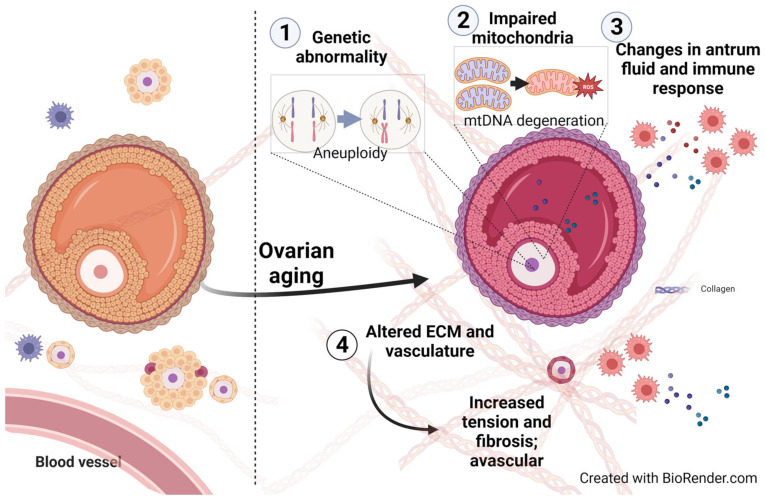
Consequences and mechanisms of ovarian aging. Ovarian aging could be partially attributed to (1) genomic abnormality in oocytes and ovarian cells, (2) impaired mitochondrial functions, (3) changes in the antrum fluid and ovarian immune landscape, and (4) altered extracellular matrix (ECM) and vascular environments.

The process of ovarian decay in aging women may be associated with (1) aneuploidy and degeneration of oocytes, (2) damage to genomic and mitochondrial DNA of oocytes and follicular cells, or (3) alterations in the ovarian environment [56,57,58] (Figure 1). Although genetic abnormalities were considered the primary factor contributing to the decline in fertility among aged women, the decline in the overall ovarian environment has also played a significant role in determining the fertility potential of women, especially those who seek IVF treatment [59]. Patients with POR tend to require longer durations and higher doses of gonadotropin stimulation during the IVF process. Additionally, POR patients tend to have lower peak levels of estradiol (E2) and fewer granulosa cells than young women with normal fertility. The situation leads to significantly lower pregnancy rates per ovarian pickup when compared to normal responders in the same age group [60,61,62].

These findings emphasize the importance of exploring strategies that improve not only the quality of oocytes but also the overall ovarian environment in the development of treatments for women facing infertility due to aging.

### 2.2. Molecular and Functional Changes in Aging Ovaries

#### 2.2.1. Genetic Abnormality

Approximately 10% of women are affected by premature ovarian aging [63]. The main cause for the high rates of oocyte and embryo aneuploidy in aged women is believed to be age-related changes in meiotic spindle formation and chromosome alignment. POI is a heterogeneous condition that affects 3.7% of women before the age of 40 years [64]. Genome-wide association analyses have identified dozens of POI-associated genes that are involved in gonadal development, meiosis, folliculogenesis, and ovulation [65], and these putative pathogenic variants could contribute to 23.5% of cases. In addition, mutations in the senataxin gene, which is involved in the repair of oxidative-stress-induced DNA damage, have been linked to POI in patients with the neurodegenerative disorder ataxia oculomotor apraxia type 2 (AOA2) and to ovarian aging in mice [66]. Additionally, the “premutation” in the fragile X mental retardation 1 (FMR1) gene has been linked to poor embryo quality and reduced chances of pregnancy in POI patients [67]. Furthermore, it was shown that women in the top 1% of genetic susceptibility to ovarian aging have an equivalent risk of POI to those carrying monogenic FMR1 premutations, which consist of 55 to 199 copies of a CGG repeat in the 5′ untranslated region [68]. These data suggested that genetic loci could play important roles in shaping the ovarian reserve and its rate of depletion during the reproductive life in susceptible patients [69]. Moreover, oocyte gene expression and genome-wide association studies have shown that various pro-longevity and anti-longevity genes, including those related to growth, metabolism, cell-cycle progression-related longevity pathways, and DNA damage response, may play important roles in regulating ovarian aging in humans [69,70]. The expression of anti-longevity genes in oocytes increases in aged patients and is negatively correlated with antral follicle count.

SNPs located in genes associated with immune and mitochondrial function as well as DNA repair and maintenance processes have been linked to early age at menopause and POI [71,72]. Among them, those involved in the regulation of DNA damage/repair and cytogenetic stability are implicated in both ovarian aging and POI [73,74,75]. Together, these data suggested that POI could share a common genetic background with ovarian aging.

The best documented effect of aging on oocytes is aneuploidy or chromosome segregation failure. The chance of aneuploidy increases by >50% in women with advanced age [76,77]. It has been suggested that chromosome segregation error related to spindle assembly checkpoint (SAC) proteins defects and loss of cohesion between sister chromatids during meiosis I could be the major factors that contribute to age-associated chromosomal segregation failures in women [76].

Advancing age can also lead to increased damage of telomeres and genomic DNA in oocytes and granulosa cells [78,79,80,81], and age-related epigenetic status has been linked to low oocyte yield and poor ovarian response in IVF patients [82,83]. Telomeres play a central role in determining cell fate and aging in every organ. A few hundred nucleotides of telomere repeats must “cap” each chromosome end to maintain genomic stability. When too many “uncapped” telomeres accumulate in aging oocytes, it may lead to genomic instability, chromosome segregation defects, aneuploidy, and apoptosis [78,79,80]. Studies of oocytes and ovarian somatic cells indicate that telomere length and telomerase activity exhibit dynamic changes during oocyte maturation and early embryo development, and that measurements of telomere length and telomerase activity in polar body, cumulus cells, and granulosa cells could help predict the developmental potential of oocytes and embryos [79]. Excessive telomere shortening may contribute to miscarriage and birth defects in aging women, perhaps due to reduced synapsis and chiasma or abnormal meiotic spindle assembly [80,84].

In addition, the expression of miRNA and long non-coding RNAs (lncRNAs) involved in the regulation of PI3K-Akt, forkhead box O (FOXO), and p53 signaling pathways in cumulus cells has been linked to the maternal age of patients [85,86]. On the other hand, dysregulated N6-methyladenosine (m^6^A) modification, which is the most prevalent epigenetic modification in mRNA, has been associated with PCOS, POI, and ovarian aging [87].

Many of these genomic and epigenetic abnormalities are presumed to occur due to the accumulation of spontaneous damage arising from increased reactive oxygen species (ROS) produced by mitochondria during normal metabolism [88]. For example, mitochondrial dysfunction has been shown to affect meiotic spindle assembly. As such, aberrant regulation of ROS production may eventually lead to spindle instability, chromosomal abnormalities, and reduced competence of aged oocytes.

Together, these findings suggest that complex interplays of genetic and epigenetic factors in both oocytes and follicular cells contribute to the aging process in the ovary and to the decline in fertility with age.

#### 2.2.2. Changes of Mitochondria in the Oocyte and Somatic Cells

The mitochondrial DNA integrity in various tissues is known to undergo alterations because of aging. The regulation of oocyte maturation, fertilization, embryo quality, and early embryo development is associated with mitochondria activity within the oocyte [89,90,91,92]. In older women, oocytes may exhibit decreased mitochondrial quality, which is characterized by decreased number of mitochondria, the accumulation of mitochondrial DNA mutations, decreased ATP production, and increased oxidative stress, leading to poor fertility or premature ovarian aging [93]. 

Granulosa cells from aging women also display an increase in mitochondrial DNA aberrations, which may reduce the defense against oxidative stress and result in an impaired NADH/NAD+ redox state in oocytes [90,94,95]. Additionally, cumulus cells from aging patients appear to activate gene pathways associated with hypoxia stress response and oxidative stress [96], while granulosa cells from aging women have a reduced level of enzymes such as superoxide dismutases and catalases [94]. As such, the decreased quality of oocytes in patients with POI or POR may be partly attributed to decreased mitochondrial activity or the accumulation of irreparable damage to follicular components due to unavoidable oxidative stress in the aging ovary [92,97]. As a result, it has been proposed that infertility in aging patients may be addressed either by restoring mitochondrial activity through the provision of nutrients or by transferring autologous or heterologous mitochondria to enhance oocyte competence [89,91,98,99]. However, whether the age-associated decline in fertility is primarily due to the aging of oocyte mitochondria is yet to be established [100].

#### 2.2.3. Altered Extracellular Matrix (ECM) in the Aging Ovary

In addition to changes within oocytes and follicular cells, the oocyte and embryo quality can be significantly influenced by alterations in the extracellular matrix (ECM) and the associated ovarian stromal compartments, which include the antrum and stromal immune cell community [101]. The interaction between oocytes and the surrounding microenvironment is facilitated by signaling molecules that diffuse through the environment and by direct contacts with the ECM and surrounding cells [102]. In the human ovarian cortex, a higher level of collagen and lower level of elastin content in the ECM surrounding primordial and primary follicles is believed to impose a high level of mechanical stress on these early-stage follicles, making the environment less permissive to the activation and growth of such follicles in prepubertal ovaries [35,103]. As the ovary ages, the structural organization of stromal components and the ovarian vasculature undergo significant changes [104,105]. There is a significant increase of the levels of collagens (COL1A1, COL3A1, COL4A2), laminins (LAMA3, LAMB1), and proteoglycans in the ECM as the ovary ages, while the levels of elastin and fibronectin decrease [103,106]. There are also shifts in the contents of microfibril interface-located protein 1 (EMILIN-1), fibrillin-1, and glycosaminoglycans that play key roles in signaling pathways related to follicle activation during development and aging. These changes are accompanied by a dynamic response in the expression of protease-related genes in aged women [106]. The protease-related genes such as ELANE, MMP1, MMP2, MMP3, MMP9, and MMP14 have significantly reduced expression in aged ovaries, whereas MMP12 transcription significantly increases in senescent ovaries.

Furthermore, ovarian aging has been associated with changes in hyaluronan matrix which is an extracellular matrix glycosaminoglycan that maintains tissue homeostasis [107]. The total hyaluronan content in ovarian stroma decreases with age, and this process is associated with increased hyaluronidase (Hyal1) and decreased hyaluronan synthase (Has3) expression [108]. In addition, it has been shown that the degraded hyaluronan product, low molecular weight hyaluronan fragments, can induce inflammatory response in ovarian stroma cell cultures [109]. Because hyaluronan is important for establishing a microenvironment conducive to normal follicle development in the ovary [110], and because hyaluronic acid can prevent immunosuppressive drug-induced ovarian damage [111], the depleted hyaluronan content in aging ovaries may play a part in destabilizing normal ovarian architecture and contribute to female reproductive aging [109].

Under normal conditions, tissue remodeling in response to injury or physiological turnover leads to tissue regeneration without permanent damage. Cyclic changes in the production and degradation of ECM are part of normal cyclic process of ovarian follicle development [112]. The ECM structure anchors and provides stability and shields cells from the environment [113]. However, as the ovary ages, the progressive accumulation of ECM and advanced glycation end-products could prevent enzymatic cleavages of the ECM necessary for normal tissue remodeling [114,115]. The dysregulated degradation and deposition of ECM renders the tissues scarred and less organized, leading to the loss of normal functions. Because tissue fibrosis is frequently accompanied by innate and adaptive inflammatory processes [116], increased fibrosis in the ovarian stroma could eventually disrupt normal folliculogenesis in patients with premature ovarian aging or POI due to enhanced tissue damage [117].

Furthermore, age-related changes in the microenvironment may damage the proper primordial follicle assembly with stem cells, and the loss of primordial follicles may itself accelerate the aging process [118,119,120,121,122].

#### 2.2.4. Alterations of Vascularization in Aging Ovaries

Studies of ovarian stromal blood flow have shown that 60% of women with low ovarian reserve have undetectable basal stromal blood flow in at least one ovary, while only 6% of women with good ovarian reserve have undetectable flow [123]. This suggests that alterations in the ovarian stromal vasculature may be associated with the pathophysiology of ovarian aging. In addition, in parallel with normal aging, ovarian tissue fibrosis was shown to be associated with blood vessel damage [124].

The ovarian stroma contains a network of blood vessels that supply nutrients, oxygen, growth factors, and various immune components to growing follicles and corpus lutea [125]. Angiogenesis is mainly active on growing follicles and corpus luteum, and it builds temporary vascular networks for each growing follicle. Vascularization is first observed in preantral follicles, and neovascularization drastically increases in tertiary follicles [126]. The degree of perifollicular vascularity of individual follicles positively correlates with the dissolved oxygen content in the follicular fluid and controls the micro-environment surrounding the developing oocyte [127]. Women who received embryos originating from oocytes developed in well-vascularized follicles had a higher pregnancy rate than women who received embryos derived from poorly vascularized follicles [128,129,130,131]. In addition, various studies have shown that progenitor/stem-cell-based therapies may improve fertility partly by increasing angiogenesis in the ovary [132,133].

Because the aging-associated endoplasmic reticulum stress could increase follicular apoptosis and the loss of ovarian vessel density [134], ovarian senescence may be partly attributed to the lack of proper blood supply in the aging ovary which is depleted of stem cells and filled with rigid ECM components [135]. However, whether the diminished basal stromal blood flow in women with low ovarian reserve is a cause or consequence of low ovarian reserve remains to be investigated.

#### 2.2.5. Shifts in Follicular Fluid Composition

The tertiary follicle’s antrum fluid serves as another dimension of the microenvironment for the developing oocyte, and its composition reflects the exchange between the oocyte and its surroundings. Follicular fluid contains various cytokines that are involved in folliculogenesis and are associated with oocyte quality and the implantation potential. Studies have shown that follicular fluid from women undergoing assisted reproductive technology (ART) treatments exhibits an age-dependent metabolomic profile [136,137]. Follicular fluid from patients with diminished ovarian reserve has a significant decrease in polyunsaturated choline plasmalogens and methyl arginine transferase activity when compared to that of patients with normal ovarian reserve [138]. Likewise, the level of platelet-derived growth factor-BB in the follicular fluid is significantly lower in patients with diminished ovarian reserve compared to those with normal ovarian reserve [139]. Studies of follicular fluid have also shown that the level of granulocyte-colony stimulating factor (G-CSF) is a predictor of oocyte competence, implantation, and ART success [140,141,142].

On the other hand, the adrenomedullin 2 peptide level in follicular fluid is significantly higher in non-responding IVF patients when compared to those in the responsive groups [143]. Similarly, the level of adrenomedullin peptide in follicular fluid is inversely correlated with the total number of oocytes retrieved from IVF patients [144]. Follicular fluid AMH levels also appear to be significantly higher in older patients compared to younger patients [53,54,55]. These findings suggest that aging may alter the accumulation of metabolites, substrates, and regulatory factors in follicular fluid. However, further research is needed to determine whether these changes are a consequence or mediator of ovarian aging.

#### 2.2.6. Alterations in the Ovarian Immune Response

The immune system has a crucial role in various aspects of female reproduction, from preparing the endometrium for blastocyst implantation to maintaining pregnancy and facilitating parturition. An altered immune response or autoimmunity such as anti-adrenal antibodies and anti-thyroid antibodies can contribute to infertility in aging women and the development of POI [6]. Studies have shown that the presence of anti-thyroid peroxidase antibodies is a risk factor for poor IVF outcome in some POR patients [145]. However, due to the lack of consensus and effective treatments for autoimmunity-mediated infertility, the exact contribution of autoimmunity to POR and POI remains to be thoroughly studied.

The aging process is associated with a general dysregulation in the immune system, known as “immune senescence” [146]. In the ovary, the aging process leads to an increase in the number of inflammatory cytokine-secreting memory T cells and the level of inflammatory cytokines such as IL-3, IL-7, IL-15, TGFβ1, TGFβ3, and MIP-1 in follicular fluid, whereas the IL-8 level in follicles is lower in older women [147,148,149]. In the ovary of aging mice, there is an age-dependent increase in NLRP3 inflammasome, and pharmacological inhibition of NLRP3 improves fertility in female mice [150]. The expression of pro-inflammatory cytokines such as IL-16 is significantly higher in ovarian stroma of women at early and late menopause as compared with premenopausal women [151]. Overall, the natural aging of the ovary is associated with the infiltration of immune cells and activation of inflammation-related signaling pathways [25].

Together, these data suggest that changes in immune cell functions in the ovarian stromal compartment could contribute to the manifestation of an aging ovarian environment [147]. This change, in turn, may decrease follicle reserve and oocyte quality in aging IVF patients [101].

## 3. Potential Treatments to Avert Infertility in Women with POR or POI: Adjunct Intervention, Gamete Engineering, and Strategies That Incorporate Mechanical Manipulation

Despite significant efforts aimed at addressing the issue of aging ovaries in IVF patients through the modification of gonadotropin stimulation regimens and methods of embryo selection, these attempts have not significantly improved the IVF clinical outcomes in most patients with POR [152]. Because the decline in fertility with age involves a range of physiological changes in the oocyte and ovarian environment, various alternative strategies have been proposed. These alternative approaches mainly aim at improving the ovarian response to gonadotropin stimulation, preventing follicular atresia prior to the ovulatory stage, or generating gametes de novo [20]. These methods can be broadly categorized into two distinct categories: adjunct intervention and gamete engineering. In addition, an emerging category that incorporates mechanical intervention has been introduced recently.

### 3.1. Adjunct Intervention

The field of pharmacological adjuvants has explored a range of hormones, steroids, cofactors, second messenger regulators, and cellular entities to enhance fertility of IVF patients. One such adjuvant is growth hormone, which has been shown to increase the density of FSH receptors, LH receptors, bone morphogenetic protein receptor type-1B (BMPR1B), and growth hormone receptors in granulosa cells of aging women with a poor ovarian reserve. Although the evidence for its efficacy in increasing pregnancy rates is weak, its potential utility in cases of extremely poor ovarian response cannot be discounted [152,153,154].

A meta-analysis of 46 trials, including 19 trials of women with POR, showed that DHEA treatment is associated with improved pregnancy rates in POR patients, whereas clomiphene or letrozole use is not recommended [155]. DHEA, which signals through multiple neurotransmitter receptors, steroid receptors, and the sigma non-opioid intracellular receptor 1, may improve ovarian reserve, oocyte quality, and pregnancy rates in women with diminished ovarian reserve or premature ovarian aging [156]. Another promising strategy for enhancing IVF outcomes is the intraovarian injection of autologous platelet-rich plasma (PRP), which has been shown to increase antral follicle count and serum AMH levels in POI patients [157,158,159,160] and restore menstruation in patients who lacked a menstrual cycle for over a year [161].

Because the accumulation of free radicals with age leads to DNA mutations, telomere shortening, and accelerated ovarian aging, the effects of several antioxidants have been studied, including melatonin and resveratrol [162]. Long-term melatonin treatment was shown to attenuate age-induced fertility decline in mice by decreasing the generation of mitochondrial reactive oxygen species and preserving respiratory chain complex activities in the ovary [15,16]. Resveratrol, with its anti-tumor and anti-aging effects through anti-oxidation and activation of Sirt1 and telomerase, has been shown to improve ovarian reserve and protect against fertility reduction in aging mice [17,18,21]. Likewise, mild calorie restriction (10–30%) has been shown to extend reproductive lifespan in aging mice [163,164].

Another area of interest is senotherapy, which uses compounds like metformin, dasatinib, and quercetin to recover ovarian functions in animals with chemotherapeutic-agents-induced ovarian injury presumably by removing senescent cells [19,21]. Senescent cells are characterized by cell cycle arrest, apoptosis resistance, and a senescence-associated secretory phenotype (SASP) [165]. Since cellular aging is the greatest contributor to ovarian aging and associated disorders, many assume that targeting cellular senescence could be a compelling strategy to extend ovarian health. Chemotherapies have been shown to increase senescent cell burden in ovarian tissues. Through the analysis of senescence-associated β-galactosidase (SA-β-gal) activity and SASP factors [166], classic senolytic agents such as dasatinib and quercetin (DQ) or fisetin were shown to reduce the load of senescent cells in ovaries of obese mice or after doxorubicin treatment [167,168]. While neither DQ nor fisetin mitigate doxorubicin-related ovarian dysfunction, senolytic treatments (e.g., metformin plus DQ) increase follicular reserve and pups per litter and reduce DNA damage in cisplatin-treated animals [19].

On the other hand, the sphingolipid ceramide-1-phosphate was shown to protect against cyclophosphamide-induced ovarian damage in a premature ovarian failure (POF) model by inhibiting apoptosis and improving stromal vasculature and oocyte quality [169]. Furthermore, a declining level of the metabolic cofactor nicotinamide adenine dinucleotide (NAD^+^) has been shown to accompany the loss of oocyte quality with age. Of interest, treatment with an NAD^+^ metabolic precursor nicotinamide mononucleotide (NMN) appeared to exert senolytic function and improve the quality of oocytes in aged animals by recovering NAD^+^ levels [170,171].

Because the intra-oocyte PI3K/mammalian target of rapamycin (mTOR) pathway plays a key role in the regulation of primordial follicle activation, the potential use of mTOR stimulators or inhibitors, such as phosphatidic acid, propranolol, and rapamycin, to prevent premature ovarian failure or promote follicle recruitment and folliculogenesis has been studied extensively [30,172,173,174,175]. Phosphatidic acid and propranolol were shown to increase the activation of primordial follicles in aged mouse ovaries and human ovarian cortex cubes [172]. On the other hand, the mTOR inhibitor rapamycin maintains the follicle pool by suppressing primordial follicle development [164]. Thus, rapamycin may have a potential for preventing premature ovarian failure, while the mTOR stimulators may promote follicle recruitment and folliculogenesis in short-term treatment regimens [164,176,177].

The use of stem cells to improve the ovarian microenvironment is another potential therapeutic approach. Studies in mice have shown that grafted human ovarian tissue with transplanted multipotent adipose-tissue-derived stem cells (ASCs) have a significantly higher follicle density compared to those without ASCs, suggesting that co-transplanted ASCs may protect primordial follicles and maintain their quiescence [178]. Mesenchymal stem cells have also been shown to enhance the ovarian follicle pool and rescue fertility outcomes in animals with radiation-induced premature ovarian failure or physiological ovarian aging [179,180,181,182]. However, the utility of stem cells in improving fertility in aging patients remains to be established through controlled studies. Overall, these adjunct treatments require further investigation in controlled settings to establish their efficacy.

### 3.2. Gamete Engineering

In addition to adjunct interventions, emerging experimental approaches include pronuclear or mitochondrial transfer and in vitro generation of gametes using pluripotent cells [12]. Recent advances have demonstrated the feasibility of reconstructing the entire process of mammalian oogenesis in vitro from primordial germ cells using an estrogen-receptor antagonist that fosters normal follicle formation and oocyte growth [183]. Additionally, mature oocytes with full potency have been generated from both embryonic stem cells and induced pluripotent stem cells derived from embryonic fibroblasts or tail-tip fibroblasts [22]. Furthermore, functional oocytes with the capacity for fertilization and development into live offspring have been derived from both pluripotent stem-cell-derived primordial-germ-cell-like cells and fetal ovarian somatic cells [23].

These advancements may enable the production of viable human oocytes in a proactive manner or as needed for individuals who may face infertility issues in the future [24]. However, it is important to note that these oocyte engineering procedures are still in the early stages of development and have yet to demonstrate a proven therapeutic effect or enter the clinical realm.

### 3.3. Strategies That Incorporate Mechanical Force for Stimulating Follicular Growth in Women with POR or POI

Advancements in the understanding of oogenesis and folliculogenesis have led to the recognition that physical interactions between follicular cells and the ECM environment also play a critical role in normal follicle progression [28,184,185]. This insight has led to the development of experimental interventions aimed at enhancing the recruitment of early follicles, partly via the regulation of mechanical interactions within the ovarian environment [26,186]. One such intervention is the In Vitro Activation (IVA) procedure, which involves the treatment of PI3K-Akt activators and mechanical disruption of the Hippo signaling pathway through ovarian cortex fragmentation [7,28,187,188].

**Figure 2:**
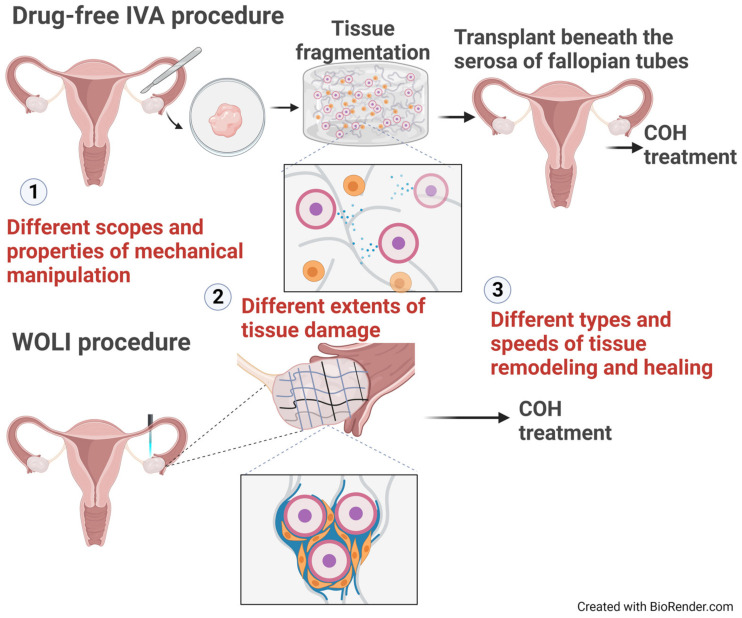
Graphical representation of differences in mechanical-force-mediated stimulation between the drug-free IVA and WOLI procedures. Drug-free IVA and WOLI procedures receive different types of mechanical manipulation, which vary in the scale and extent of tissue damage surrounding ovarian follicles and presumably the scope and speed of tissue regeneration in ovarian stroma. As such, they may lead to different patterns of follicle recruitment and growth. The orange cells in insets represent granulosa cells that surround the oocytes.

Another experimental approach is the WOLI/LOI procedure, which aims to break the restrictive environment imposed on resting follicles using simple mechanical manipulation in vivo [36,37]. While these interventions remain in the experimental stage, they represent a promising and practical avenue for enhancing fertility in POI and POR patients (Figure 2).

#### 3.3.1. IVA: An Evolving Experimental Technique Involving Pharmacological Stimulation and Mechanical Manipulation

IVA is a pioneering procedure that involves the cultivation of mechanically separated ovarian fragments with different pharmacological stimulations and the subsequent autotransplantation in patients [3,7,175,187,189,190]. Orthotropic transplantation of ovarian strips treated with the mTOR stimulator phosphatidic acid and the PI3K stimulator 740Y-P was shown to produce new waves of follicle growth and live births in women with POI [189]. Additionally, the combination of IVA procedure and autologous bone marrow mesenchymal stem cell transplantation has been shown to improve follicle growth and oocyte aspiration in women with premature ovarian failure [191]. In the IVA process, the mechanical fragmentation process disrupts the Hippo signaling pathway which normally regulates cell proliferation by inhibiting yes-associated protein (YAP), transcriptional coactivator with PDZ-binding motif (TAZ), and coactivators of the Scalloped (Sd or TEAD) in ovarian tissues [192]. The combination of mechanical and pharmacological stimulation also activates pathways that involve YAP, TAZ, PTEN, mTOR, and forkhead box O3 (FOXO3) in primordial and primary follicles [38,175,193,194,195,196,197]. Mechanically, the IVA process was shown to increase actin polymerization and trigger YAP nuclear localization, which then upregulates downstream targets such as BIRC1 and CCN2 and eventually results in improved follicle activation and growth [187,198].

A review of outcomes of different IVA procedures from 2013 to 2019 showed that, while women with POI have a 5% chance of spontaneous pregnancy, the IVA method allows a 30% pregnancy rate [38,199]. This suggests that, through modulation of the Hippo and PI3K/Akt signaling pathways, the IVA procedure is an effective approach to improve primordial follicle activation in POI and POR patients [7,29,30].

#### 3.3.2. Drug-Free IVA: Mechanical Force Alone Is Sufficient to Activate Dormant Follicles

Soon after the introduction of the original IVA procedure, a drug-free IVA technique garnered attention in the field. This technique utilizes pure mechanical manipulation in the form of ovarian cortex fragmentation to stimulate follicle growth by promoting actin polymerization and the translocation of YAP [38,190,199,200,201]. The auto-transplantation of fragmented ovarian cortical tissue was shown to increase antral follicle counts and result in a high fertilization rate and live birth in patients with POI, POR, or diminished ovarian reserve [38]. This approach could be safer and time-saving because it does not require the use of pharmacological agents and could be accomplished with just one surgical procedure [190]. However, the efficacy of the drug-free IVA procedure remains uncertain, as it has not been proven in controlled studies [202,203]. Additionally, the amount of damage applied to the small ovarian cortex pieces during fragmentation is difficult to control, and studies have indicated that the number of surviving follicles in fragmented cortex tissues could be lower compared to intact cortex tissues [204,205,206]. Therefore, further research and controlled studies are necessary to determine the safety and efficacy of this simplified IVA procedure.

#### 3.3.3. Whole Ovary Laparoscopic Incision (WOLI) Procedure: Rescuing Aging Ovaries via Surgical Intervention

One approach to circumvent the drawbacks of ovarian fragmentation during IVA is to apply mechanical manipulation in vivo through a series of surgical incisions on ovaries. Recent studies have shown that a minimally invasive laparoscopic incision-based procedure, known as whole ovary laparoscopic incision (WOLI or LOI), can restore hormonal responses in aging patients with extremely poor ovarian response (EPOR) and those with resistant ovary syndrome (or Savage syndrome) who present with hypergonadotropic hypoestrogenism, similar to that seen in patients with POI [28,36]. These findings were corroborated by the study of gc*Nrg1*KO mice, which showed that surgical cutting of ovarian surface reduces ovarian fibrosis and increases the vascular supply to follicles in aging mice, leading to the restoration of responses to exogenous hormones and the estrous cycle, with fertility maintained for three months [207].

The WOLI procedure is performed as an outpatient procedure and involves making parallel streak incisions using a scalpel blade, with a space of 1–2 mm between them and a depth of 2–3 mm [36]. Parallel streak incisions were first made on one side of the ovary. After completing the first set of parallel incisions, perpendicular incisions with the same depth and distance were made. This design creates extensive mechanical disturbance in ECM but has minimal damage on the overall structural integrity of ovarian tissues. Once the operation is completed, the whole ovary is dressed with a fibrin sealant to minimize bleeding.

Most patients showed small antral follicle growth as soon as the fifth day after surgery. Once growing antral follicles were detected, natural or a mild stimulation cycle was applied. In cases with gonadotropin stimulation, oocytes were retrieved at 35–36 h after ovulation trigger, followed by frozen–thawed embryo transfer cycle. After the treatment, five out of six EPOR patients had significant increases in serum estradiol level and improved follicle growth, and it led to thawed embryo transfer cycles in four patients.

This approach aims to create mechanical disturbance in the ECM and expose resting follicles to similar changes in mechanical tension, while preserving the overall structural integrity. By mechanically altering mechanical tension within tissues, the WOLI procedure manipulates the mechano-sensing process and disrupts environment-associated biochemical signals. It minimizes damage to the remaining follicle pool and eliminates the need for auto-transplanting ovarian cortex fragments into tissue pockets.

Overall, these findings suggest that (1) mechanical forces within the ECM environment are a crucial factor in determining follicle fate, (2) the ovarian ECM-associated Hippo/YAP signaling pathway represents a prime target for improving follicle growth, and (3) surgical procedure alone is sufficient for improving the growth of remaining follicles that are normally destined for demise in POR patients. While the exact mechanisms that underlie IVA- and WOLI-mediated stimulation of folliculogenesis remains to be fully vetted, it likely involves an array of physiological pathways that ultimately generate an accommodating environment suitable for the continuing growth of remaining follicles in patients. This array of physiological processes could include (1) alteration of mechanobiological signaling in the ECM environment, (2) disruption of the hypoxic microenvironment and the fibrotic signal barrier, and (3) stimulation of angiogenesis and aseptic inflammation responses.

#### 3.3.4. Ovarian Surface Cutting Improves Follicular Development in Aging gc*Nrg*1KO Mice

In parallel with the exploration of IVA procedure for fertility improvement in POR and POI patients, studies of gc*Nrg*1KO mice, in which the expression of neuregulin 1 (*Nrg*1) gene in granulosa cells of female mice is disrupted by the *Nrg*1^flox/flox^/Cyp19-Cre transgenes, have provided a rare glimpse of how ovarian aging affects ovarian stromal environment and follicle development and how mechanical manipulation may affect this process.

Neuregulin 1 is an epidermal growth factor family protein that acts on the EGFR family of receptors. It is essential for the development of cardiac and nervous systems and the maintenance of normal heart structure [208]. In addition, the loss of Nrg1 within cortical neurons was shown to reduce synaptic plasticity [209]. In the ovary, Nrg1 expression in granulosa cells is induced by LH. Studies using Nrg1-knockdown and exogenous Nrg1 treatment showed the gonadotropins-dependent Nrg1 signaling in granulosa cells is important for supporting follicular growth and oocyte meiotic maturation [210]. Consistently, targeted disruption of *Nrg*1 in granulosa cells of gc*Nrg*1KO mice reduces the number of pups per litter, perhaps due to aberrant regulation of protein kinase C signaling in cumulus cells [211,212]. Studies of these mice also indicated that the loss of Nrg1 expression in granulosa cells accelerates reproductive aging [212].

The aging female gc*Nrg*1KO mice have ovarian and endocrine phenotypes like that of aging women or low responders. The 6-month-old gc*Nrg*1KO mice have high circulating levels of FSH, low levels of AMH, abnormal estrous cycle, and reduced number of ovulated oocytes. In these mice, folliculogenesis is arrested in the secondary follicle stage, and the ovarian stroma is enriched with heterogeneous LH receptor-positive endocrine cells and actin-rich fibrotic cells [212]. When the LH level is suppressed in these mice, it leads to the disappearance of fibrotic cells and the resumption of follicle development and estrous cycles. These data suggest that Nrg1 is not only important for normal progression of folliculogenesis during the reproductive life but also plays a critical role in the regulation of ovarian stromal environment. The life-long deficiency of Nrg1 in the ovarian microenvironment increases ovarian fibrosis, and this, in turn, adversely impacts normal folliculogenesis in aging mice.

Because the ovarian stroma compartment in POR patients and aging mice are often fibrotic and avascular, and because neovascularization near the follicles increases dramatically as ovarian follicles mature [213,214], it has been proposed that insufficient supply of FSH resulting from the avascular and fibrotic ovarian environment may contribute to follicular arrest in aging gc*Nrg1*KO mice [212]. In addition, it was hypothesized that cutting the ovarian surface in aging gc*Nrg1*KO mice may relieve the fibrotic stress and improve perifollicular circulation and, hence, follicle growth [207]. Indeed, ovarian surface cutting in aging gc*Nrg1*KO mice reduced ovarian fibrosis and induced vascular formation near the follicles 7 days after surgery. The estrous cycle and the response to exogenous hormones recovered 21 days after the surgery in these mice, resembling what occurs after the WOLI and LOI procedures in patients.

Because decreased tissue fibrosis occurs in parallel with the recovery of folliculogenesis after ovarian surface cutting, and because these two processes are reversable, it is reasonable to assume that (1) Nrg1 deficiency-mediated ovarian aging is particularly associated with alterations in the ovarian stromal compartment and (2) the procedure may relieve secondary follicles from the rigid environment partly via the mechano-signaling pathways or by initiating a tissue remodeling process that reduces stress fibers and collagen accumulation in ovarian tissues. Alternatively, the recovered follicular growth in these mice could be a result of improved blood flow and/or supply of FSH to the follicles, like that which occurs after the laparoscopic ovarian drilling in PCOS patients [215].

While Nrg1 deficiency is unlikely a cause of reduced fertility in POR and POI patients, studies of these mice have demonstrated that the fibrosis status of the ovarian stromal environment is closely associated with the ability of secondary follicles to develop further.

## 4. Potential Mechanisms That Underlie Mechanical-Manipulation-Mediated Stimulation of Folliculogenesis

### 4.1. Changes of Mechanobiological Signaling in the ECM Environment

It is known that cells can sense and generate mechanical force, which could lead to physical property-induced biochemical changes and intracellular signaling [198,216,217,218,219,220]. This in turn can modulate nuclear events and regulate morphogenesis and pattern formation. The process of folliculogenesis in mammals is influenced by interactions between mechanical forces and various components within the ovary. As such, the IVA and WOLI procedures may facilitate follicle growth partly by modulating such interactions.

The primordial follicle begins with the aggregation of a single layer of granulosa cells surrounding the oocyte. As the follicle progresses to the primary stage, it displays cuboidal granulosa cells and a zona pellucida (ZP) encapsulating the oocyte. The secondary follicle stage is characterized by an increased number of granulosa cell layers and the presence of an outer theca cell layer. Under the influence of gonadotropins, the secondary follicle gradually grows to a preovulatory follicle of ~20 mm while the theca wall undergoes extensive ECM remodeling and the antrum expands. Changes in mechanical tension in the antrum and follicular wall, in conjunction with enzymatic remodeling, are believed to play crucial roles in modulating follicle rupture during ovulation [221]. This makes the whole folliculogenesis process itself a mechanosensitive development course [193,221]. The mechanical environment around the oocyte at the early stage of follicle development could involve various intra-follicular and extra-follicular ECM interactions [222]. The basal lamina, granulosa cells, theca cells, and stromal cells could all play a role in this environment [223,224]. For example, granulosa cells may secrete mucopolysaccharides that thicken and stiffen the zona pellucida and contribute to follicle dormancy, while theca cells provide structural support and produce endocrine regulatory factors [225]. On the other hand, the stiff basal lamina may alter the mechanical tension around the oocyte and affect molecule passage [103].

**Figure 3:**
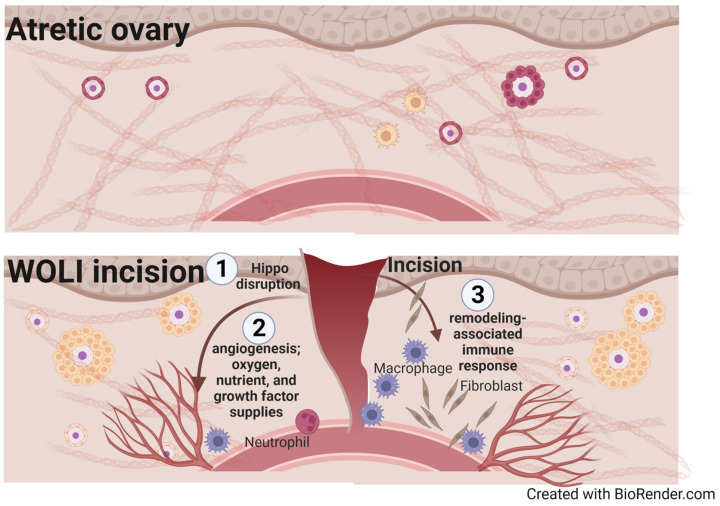
Graphical representation of potential mechanisms that contribute to the WOLI-mediated follicle growth. The WOLI procedure could lead to (1) disruption of the Hippo signaling pathway, (2) stimulation of angiogenic pathways, increased supply of oxygen, nutrients, and various growth factors, and (3) tissue remodeling and associated immune responses, which altogether help rejuvenate the ovarian environment in an atretic ovary. Growing follicles are indicated by the presence of replicating granulosa cells (i.e., yellow cells surrounding the oocytes).

The mechanical stress imposed by the collagen-rich and avascular ECM surrounding the follicles in the ovarian cortex is presumed to be important for maintaining the dormant state of primordial follicles by modulating granulosa cell communication [103,105,221,226,227,228,229,230,231]. It is also believed that physical compression of primordial follicles may occur through interactions across cell–cell junctions and the surrounding ECM. The activation of quiescent primordial follicles may be initiated partly by altering Hippo signaling, which can respond to various stimuli such as ECM stiffness, stretching, cell geometry, cell density, cellular tension, and shear stress [222] (Figure 3). Consistent with this idea, the loosening of ovarian cortex structure was shown to activate dormant oocytes while the dormancy can be restored by increasing compression with exogenous pressure [35,232].

As women age, the total content of hyaluronic acid decreases while the overall collagen deposition increases. This leads to increased fibrosis and tissue stiffness in the ovary, like other organs such as the heart, lungs, and kidneys [107,228,233,234]. This fibrotic ECM environment can place additional mechanical stress on both dormant and growing follicles, making the ovarian environment inhospitable to the recruitment and growth of follicles [107,226,228,235]. Therefore, the WOLI and drug-free IVA processes may physically break this inhospitable mechanical restraint and switch the signaling status in the ovary to a more “normal” state for further development of remaining follicles [28,185,187,201,217,236,237,238,239,240] (Figure 3).

The use of mechanical manipulations to promote follicle development is not a new concept. Physical interventions, such as ovarian cystectomy, ovarian wedge resection, and laparoscopic ovarian drilling, have been used to counteract the stiffer and aberrant ovarian environment in women with clomiphene-resistant PCOS [241,242,243,244,245]. These interventions were shown to improve fertility in PCOS patients perhaps by reducing the AMH level or inducing local inflammatory reactions that are important for promoting ovulation [244,246,247].

### 4.2. Disruption of the Hypoxic Microenvironment and the Fibrotic Signal Barrier

In addition to compressive mechanical force, the stiff microenvironment surrounding immature follicles can restrict the access of oxygen, nutrients, substrates, regulatory factors, and stromal cells to the follicle. Incisions adjacent to a follicle could break down the layers of stiff ECM that hinder the exchange of oxygen and metabolites, and the flow of nutrients and regulators essential for proper follicle recruitment and growth [248,249,250,251].

The distribution of these supplies is primarily carried by the vasculature and then through passive diffusion or active transport across the vascular barrier and into the interstitial and tissue compartments. The efficiency of this process is governed by factors such as the size, charge, pKa, and lipid/water partition coefficient of the molecules. Small molecules like nutrients have a large volume of distribution and can easily reach their target cells, while large proteins like growth factors have a smaller volume of distribution and are mainly confined to the intravascular fluid. In aging ovaries, the dense ECM barrier may reduce the “volume of distribution” of different growth factors and gonadotropins, and the maximum concentration that eventually reaches the oocyte and follicular cells.

This scenario is supported by a study of reproductively aged female mice (i.e., 9-month-old retired breeders maintained until 12 to 16 months old) in which several pharmacological agents that modulate tissue fibrosis, mitochondrial function, and/or cell growth were used to interrogate the roles of fibrosis in ovarian aging [228]. These pharmacological agents included pirfenidone (an anti-inflammatory/antioxidant agent), nintedanib (an inhibitor of tyrosine kinases such as VEGFR, FGFR, and PDGFR), BGP-15 (an insulin sensitizer that can improve mitochondrial function), metformin (an activator of AMP-activated protein kinase, which can normalize mitochondrial function and suppress tissue fibrosis), MitoQ (a mitochondria-targeted CoQ10 ubiquinone), and rotenone (an inhibitor of the mitochondrial respiratory chain). Among them, antifibrosis drugs, pirfenidone and BGP-15, were shown to reduce collagen and mitochondria dysfunction and restore folliculogenesis and ovulation in aging mice [228]. Overall, the combined results suggested that impaired oocyte growth in aged mice could be partly attributed to ovarian fibrosis, which is initiated by disruption of mitochondrial bioenergetics and oxidative damage within the ovarian stroma. Furthermore, the study showed that (1) the expression of cytokine *Tgfb1,* known to promote tissue fibrosis, in the ovarian stoma is increased by aging and reduced by BGP-15, and (2) BGP-15 may reduce ovarian fibrosis partly by enhancing matrix metalloproteinase 13 (MMP13) expression. Moreover, these studies raised the possibility that select anti-fibrotic drugs could be useful for improving fertility in aging women.

As such, the WOLI procedure may enhance the chance of survival and growth of follicles by exposing them to (1) a fresh oxygenated environment and a better supply of stimulatory factors and (2) a less restrained stromal environment.

In addition, because ECM itself can function as a reservoir for various regulatory factors, a shift in the ECM composition in aging ovaries may alter the affinity and holding capacity for different signaling factors that are essential for normal folliculogenesis [252,253]. However, whether the WOLI and IVA procedures affect this balance in ovarian tissues remains to be explored.

### 4.3. Stimulation of Angiogenesis and Aseptic Inflammation

The WOLI and LOI procedures involve physical separation of tissues, which is expected to result in a significant reactive wound healing and tissue remodeling response within the ovary. The amount of ovarian stromal vascularity has been positively correlated with antral follicle count, retrieved oocytes, and pregnancy rates in IVF patients [44,123,214]. In addition, human ovarian grafts co-transplanted with exogenous endothelial cells, which form functional blood vessels at the graft interface, have been shown to improve antral follicle development, perhaps by improving perfusion to the transplanted tissue [254,255]. On the other hand, the disruption of *Vegfa* and the injection of VEGF inhibitors decrease the number of ovulated oocytes during the hormone treatment cycle in mice [214]. As such, the reparative response following the WOLI injury may increase the expression of angiogenic factors and various enzymes that are involved in vascular remodeling at the wound edge to facilitate folliculogenesis [256]. Although which groups of factors/enzymes are involved in the angiogenic and tissue remodeling response after WOLI procedure in patients remain to be characterized, studies of aging mice indicate that proteases such as MMP13 may play a role [228].

Additionally, mechanical incisions could initiate a significant aseptic inflammatory response, which would attract a plethora of immune cells to the wound site to promote healing. Changes in the mechanical and physical properties of the ECM have been shown to impact the function, polarization, and migration of macrophages [257]. The WOLI procedure may thus accelerate follicle growth partly by fostering the injury-associated angiogenesis, lymphangiogenesis, and aseptic inflammatory responses resembling those occur prior to and during the ovulatory process [240,258,259]. In support of this hypothesis, studies of reproductively aged female mice have shown that the expression of various inflammatory chemokines such as *Ccl2*, *Ccl3*, *Cxcl2*, *Il4, Il6,* and *Il13*, and *Tnfa* is increased in the ovarian stroma of aged mice, and the expression can be modulated by antifibrotic drug BGP-15.

It is also important to note that, while all the above-mentioned mechanisms could contribute to the outcomes after ovarian surface cutting in gc*Nrg1*KO mice and the WOLI procedure in EPOR patients, the extent of involvement of these mechanisms could differ in these two procedures. With a life-long deficiency of Nrg1 background, many ovarian processes in aging gc*Nrg1*KO mice could have deteriorated significantly and be very different from that of aging POR and POI patients.

Overall, these data highlight the potential and logic of using mechanical manipulations to modify the fate of resting follicles in patients. Although it is difficult to determine the exact contribution of Hippo signaling disruption, injury-induced tissue remodeling, and angiogenesis/inflammatory responses to the mechanical injury-facilitated follicle growth, current evidence suggests these physiological consequences likely work together or in tandem to improve follicle growth in an environment that is often atretic and avascular in patients with POR. However, a scenario in which one of these factors represents the driving force for recovering folliculogenesis after the WOLI procedure while the rest of these processes constitute collateral events cannot be excluded.

Despite the potential benefits, it remains a challenge to accurately quantify the mechanical force that is needed to exert a desired medical effect in POI and POR patients and to compare the efficacy of different procedures in a controlled manner. Further investigation of treatment protocols and the molecular and medical consequences in patients is needed to reveal the mechanisms of action of these mechanical procedures.

## 5. WOLI vs. Drug-Free IVA: Potential Differences in Their Actions and Physiological Consequences

WOLI and drug-free IVA procedures have demonstrated that mechanical manipulation itself is sufficient to facilitate follicle growth in patients with a depleted ovarian environment. As only two studies have reported the WOLI/LOI procedure in patients [36,37], it is impossible to evaluate which of these procedures is advantageous. However, there are indications that WOLI and drug-free IVA differ in their effects on folliculogenesis. The improved hormonal responses seen after the WOLI surgery occur within weeks, rather than months after the IVA procedure in most patients. Earlier studies of cryopreserved ovarian tissues have shown that the mean time from ovarian tissue transplantation to the restoration of hormonal response is approximately 3–4 months. As such, it normally takes longer than 3 months for a human primordial follicle to become a mature one [260,261]. Because the time between the WOLI surgery and oocyte retrieval in EPOR patients is ~24 days, the WOLI procedure most likely acts by rescuing developing secondary follicles that would otherwise become atretic. On the other hand, the IVA procedure, in which oocytes are retrieved months after the procedure, could mainly work by activating dormant primordial follicles [7,187,190]. These procedures may therefore have different effects on the development paths of primordial, primary, and secondary follicles. This difference in follicle recruitment pattern may be associated with several different aspects of these two mechanical procedures.

First, it could be related to the scale of mechanical manipulation these procedures provide. In the IVA procedure, mechanical stimulation is limited to the ovarian portion that is biopsied and processed for transplantation. In contrast, the WOLI procedure applies mechanical force to the entire ovary. As the mechanical signal is not transmitted through diffusion, the WOLI procedure may alter tensions within all layers of ovarian tissue and prevent the remaining growing follicles from failing the folliculogenesis process. While the WOLI procedure may also stimulate the recruitment of early follicles in the process, its action on secondary follicles could be the most apparent results after gonadotropin stimulation. As such, the mechano-biochemical loops that occur after the WOLI surgery could be more effective in rescuing secondary follicles compared to the IVA procedure. Consequently, the WOLI procedure may have the advantage of reducing the waiting time to the pickup of mature oocytes after the procedure.

Second, the difference in follicle recruitment pattern could be linked to the degree of damage to the remaining follicle pool. The WOLI surgery allows for the delivery of a more controlled amount of mechanical manipulation and mainly results in macroscopic tissue detachment. As such, it could have minimal damaging impact on both dormant and growing follicles. On the other hand, the in vitro fragmentation process used in the IVA procedure could result in severe tearing and abrasion of stromal compartments and cause physical damage to most remaining follicles except for the smallest ones. As such, most oocytes retrieved after the IVA procedure were obtained months after ovarian tissue auto-transplantation.

Third, differences in the scale and speed of healing reactions could also be associated with the pattern of follicle recruitment of these two procedures. Tissue injury is a natural mechanism for recalling tissue growth and rejuvenating damaged tissues. While both procedures may induce tissue remodeling like that occurs during each follicle growth cycle [185], the more extensive damage following the IVA procedure may render the revascularization process less efficient and lengthier, thereby making the reorganized ovarian environment less hospital to the recruitment and growth of follicles. As such, it takes longer to obtain a mature follicle when compared to the WOLI procedure.

These propositions are supported by studies of aging rodents. Studies of ovarian surface cutting in aging ovaries of gc*Nrg1*KO mice showed that, seven days after surgery, the fibrotic area is significantly decreased and both follicles and corpus lutea are fluently observed, presumably resulting from the recruitment of secondary follicles [207,212]. Thus, there could be fundamental differences in the mechanism and follicle recruitment pattern between the WOLI and IVA procedures.

## 6. Ways to Improve and Validate the WOLI Procedure

The success of mechanical procedures for improving fertility in select IVF patients has raised the hope of further advancement in this area; however, the power of these uncontrolled studies is quite limited. A better understanding of the underlying mechanisms of these procedures is needed to advance them for expanded application. This will require further investigation into (1) why remaining follicles in patients with POR or POI remain dormant or fail to develop under controlled ovarian hyperstimulation conditions and (2) how mechanical procedures improve follicular development. Additionally, because substrate topography and stiffness influence cell viscoelastic behavior [220], it would be interesting to examine how the depth of incisions affects the outcome and whether the WOLI procedure should be performed at a specific stage of the menstrual cycle. It is also important to investigate whether a unilateral surgical approach is a valid controlled design in future studies because the WOLI surgery may give rise to systemic effects. Likewise, questions like “what is the optimal interval for repeating the procedure in the same patient if needed?” should be answered. In addition, future studies should prioritize patients who have a limited hope of achieving pregnancy through the standard IVF treatment due to the invasive nature of the WOLI procedure [262]. Moreover, because studies of rodents have implicated the involvement of various factors such as mitochondrial bioenergetics, MMPs, and cytokines in the rejuvenation of aged ovaries after pharmacological treatments [207,212,228], it would be interesting to evaluate the status of corresponding factors in the ovary of patients after the WOLI procedure and determine whether the WOLI procedure reduces stromal fibrosis or mitochondrial dysfunction in the ovary.

By addressing these questions and concerns in randomized controlled trials, we can gain a better understanding of the pros and cons of the WOLI procedure for extending the reproductive life of patients with POR or POI.

## 7. Perspectives and Future Directions

The decline of fertility in patients with POR or POI could be a result of complex and multifaceted ovarian aging process. Ovarian aging could result from changes within the oocytes, follicular cells, and the ECM and ovarian stromal compartments. Advances in our understanding of mechanobiology during folliculogenesis have provided valuable insight for developing novel strategies such as IVA and WOLI to better manage infertility in patients with POR or POI. Among these procedures, WOLI represents a promising and practical avenue to improve reproductive outcomes in infertile women who have run out of existing options to conceive, as well as to study the underlying mechanisms. Some important aspects to consider for optimizing this procedure include the timing of the surgery, the optimal depth of incisions, the ideal interval between separate WOLI procedures, and the choice of a suitable control group. Additionally, future studies should focus on patients who have the least hope of achieving pregnancy through conventional IVF procedures. With proper validation in future controlled studies, the WOLI-like procedure could become a valuable tool for helping aging women to conceive. We also hope that the scientific community will continue to direct efforts towards the optimization of innovative mechanical approaches for improving fertility treatment.

## Data Availability

All data are presented in the figures and tables of this study.
